# Sound Vibration-Triggered Epigenetic Modulation Induces Plant Root Immunity Against *Ralstonia solanacearum*

**DOI:** 10.3389/fmicb.2020.01978

**Published:** 2020-08-21

**Authors:** Jihye Jung, Seon-Kyu Kim, Sung-Hee Jung, Mi-Jeong Jeong, Choong-Min Ryu

**Affiliations:** ^1^Molecular Phytobacteriology Laboratory, Korea Research Institute of Bioscience and Biotechnology (KRIBB), Daejeon, South Korea; ^2^Department of Biological Sciences, Korea Advanced Institute of Science and Technology (KAIST), Daejeon, South Korea; ^3^Personalized Genomic Medicine Research Center, Korea Research Institute of Bioscience and Biotechnology (KRIBB), Daejeon, South Korea; ^4^Biosystems and Bioengineering Program, University of Science and Technology, Daejeon, South Korea; ^5^National Institute of Agricultural Science, Rural Development Administration, Wanju, South Korea

**Keywords:** epigenetics, ChIP-seq, miRNA-seq, RNA-seq, sound vibration, plant immunity, *Ralstonia solanacearum*

## Abstract

Sound vibration (SV) is one of the several environmental stimuli that induce physiological changes in plants including changes in plant immunity. Immune activation is a complicated process involving epigenetic modifications, however, SV-induced epigenetic modifications remain unexplored. Here, we performed an integrative analysis comprising chromatin immunoprecipitation (ChIP) and microRNA sequencing (miRNA-seq) to understand the role of SV-mediated epigenetic modifications in immune activation in *Arabidopsis thaliana* against the root pathogen *Ralstonia solanacearum*. Plants exposed to SV (10 kHz) showed abundant H3K27me3 modification in the promoter regions of aliphatic glucosinolate biosynthesis and cytokinin signaling genes, leading to transcriptional changes that promote immunity. Additionally, 10 kHz SV down-regulated *miR397b* expression, thus activating three target *LACCASE* transcripts that mediate cell wall reinforcement via lignin accumulation. Taken together, SV triggers epigenetic modification of genes involved in secondary metabolite biosynthesis, defense hormone signaling, and pre-formed defense in *A. thaliana*, leading to the activation of plant immunity against *R. solanacearum*.

## Introduction

Being sessile, plants have to withstand various harsh environmental conditions such as wind, rain, and pathogen invasion (Basu and Haswell, 2017). Sound vibration (SV) is one of the natural stimuli that induce physiological changes in plants (Jung et al., 2018). Recent studies show that SV increases disease resistance in plants. In *Arabidopsis thaliana*, it has been shown that the chewing sound of an insect causes increased production of plant immunity-related chemicals such as glucosinolates (GSs) and anthocyanins (Appel and Cocroft, 2014). In addition, exposure of *Arabidopsis* plants to 500 Hz of SV has been shown to increase the production of plant defense-related hormones such as salicylic acid and jasmonic acid (Ghosh et al., 2016). In tomato (*Solanum lycopersicum*), 0.08–2 kHz SV treatment decreases the population of multiple pests and pathogens, including spider mites, aphids, viruses, and gray mold, in the greenhouse (Tianzhen et al., 2009; Hassanien et al., 2014).

Besides physiological changes, SV also causes transcriptional changes in genes involved in plant immunity. Recently, microarray analysis suggested that *Arabidopsis* plants pre-exposed to 1 kHz SV showed induced resistance against *Botrytis cinerea* and transcriptional changes in defense-related genes (Choi et al., 2017). However, studies investigating the effect of SV on plant immunity are generally limited to insects and fungi, and lack research on the transcriptome. Enhancement of the plant immune system via transcriptional changes involves a complex epigenetic regulatory network comprising modification of chromatin (histone proteins and DNA) and modulation of small RNAs [short interfering RNAs (siRNAs) and microRNAs (miRNAs)] (Vaucheret, 2006; Kouzarides, 2007; Kaikkonen et al., 2011; Gan et al., 2013; Catalanotto et al., 2016). Many studies have investigated epigenetic changes in defense-related genes due to histone modifications (Mengel et al., 2017; Ramirez-Prado et al., 2018b). For instance, hyperacetylation of histone H3 lysine (K) residue at amino acid position 9 or 14 (H3K9/14) activates stress-responsive genes in *Arabidopsis* (Mengel et al., 2017). Similarly, H3K9 acetylation (H3K9ac) induces the expression of defense-related genes such as *PR1*, *WRKY46*, and *WRKY53* (Ramirez-Prado et al., 2018a). Therefore, chromatin immunoprecipitation (ChIP) assay using specific histone modification markers could help to understand the epigenetic mechanisms underlying defense-related gene regulation (Park, 2009).

Gene transcription is also regulated by miRNAs (Vaucheret, 2006). For example, *miR393* and *miR160* promote immunity against *Pseudomonas syringae* pv. tomato DC3000 in *Arabidopsis* by suppressing the expression of genes encoding F-box auxin receptors and auxin response factors (ARFs), respectively. Nucleotide-binding site leucine-rich repeat (NBS-LRR) receptor proteins, another component of the plant innate immune system, recognize species-specific pathogen effectors, resulting in effector-triggered immunity (ETI) (Jones and Dangl, 2006; Zhang et al., 2019). Notably, miRNA-modulated *NBS-LRR* genes have been reported in various plant species such as *Arabidopsis*, alfalfa (*Medicago truncatula*), peanut (*Arachis hypogea*), tobacco (*Nicotiana tabacum*), and tomato (Zhai et al., 2011; Boccara et al., 2014; Zhang et al., 2019).

The soil-borne root pathogen, *Ralstonia solanacearum*, infects more than 200 plant species worldwide, causing enormous losses in crop yield (Lowe-Power et al., 2016; Lopes and Rossato, 2018). *R. solanacearum* enters the plant through root hairs and colonizes the stem tissue. Exopolysaccharides secreted by *R. solanacearum* directly cause wilting by physically blocking water flow in the densely colonized xylem vessels of the infected host. To date, no effective control strategies have been developed to control the spread of *R. solanacearum*. In the current study, we attempted to assess the epigenetic changes during SV-mediated immunity in plant roots. Although epigenetic changes in plant immunity are important, the effect of SV on these changes has not yet been reported. Here, we identified a specific frequency of SV that increases plant immunity against *R. solanacearum*. To understand the regulatory mechanisms of epigenetic changes required for the activation of plant immunity, we employed ChIP-seq, RNA-seq, and miRNA-seq. Our results provide strong evidence showing that SV is an effective physical trigger to induce resistance against *R. solanacearum* via epigenetic regulation of secondary metabolites and defense hormones, leading to SV-activated defenses. This is the first report of SV-mediated epigenetic modification of plant immunity-related genes.

## Materials and Methods

### Plant Material and SV Treatment

**FIGURE 1:**
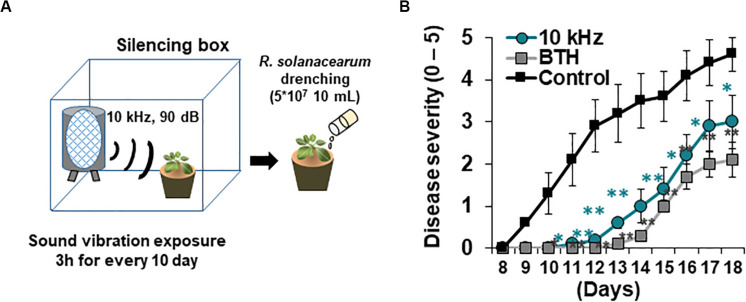
Elicitation of induced resistance by 10 kHz sound vibration (SV) application in *Arabidopsis thaliana* plants against a soil-borne bacterial pathogen, *Ralstonia solanacearum*. **(A)** Schematic representation of SV treatment and *R. solanacearum* GMI1000 inoculation. **(B)** Disease severity in SV (10 kHz)-treated, benzothiadiazole (BTH)-treated, and control plants during disease progression. Disease severity was scored on a scale of 0 (no symptoms) to 5 (complete plant collapse). Data represent mean ± standard error (SE; *n* = 10; *p < 0.05; **p < 0.01).

*Arabidopsis thaliana* ecotype Columbia (Col-0) and *dcl2/4*, *rdr2-1/6-15*, *cyp79, cyp83, sur1, ahk2, ahk2/3*, and *cre1* mutants were used in this study. Plants were grown in pots containing soil at 23°C under a 15 h light/9 h dark photoperiod. After 12–14 days, plants were transferred to a plant culture incubator (23°C, 15 h light/9 h dark) for SV treatment. Plants were exposed to SV (0.2, 1, 5, 10, 15, and 20 kHz) at 90 dB every 3 h for 10 days using the Pro Tools M-Powered software (Avid Technology, Burlington, MA, United States) (Figure 1A). Background noise was ∼40 dB in the silencing box. After inoculation with *Ralstonia solanacearum*, plants were transferred to a plant culture incubator maintained at 30°C and a 12 h light/12 h dark photoperiod for monitoring disease progression.

### Pathogen Inoculation and Disease Severity Assay

*R. solanacearum* GMI1000 was cultured on CPG (1 g/L casein, 10 g/L peptone, and 5 g/L glucose) agar medium for 1 day. A colony was picked and cultured in CPG broth for 8–10 h. A 2% volume of seed culture was inoculated in CPG broth and cultured for 20 h. The culture was then centrifuged at 8,000 rpm for 10 min. The pellet was resuspended in 10 mM MgCl_2_ prepared in sterilized distilled water (SDW). Each plant was drenched with 10 mL (5 × 10^7^ cells/mL) of the pathogen suspension. Disease severity was measured daily on a scale from 0 to 5 (0, no symptoms; 1, < 50% of rosette leaves wilted; 2, < 100% of rosette leaves wilted; 3, 100% of rosette leaves wilted; 4, 100% of rosette leaves wilted and stems partially wilted; 5, complete plant collapse). A drench application of 10 mL 0.3 mM benzothiadiazole (BTH) 3 days before pathogen inoculation was used as a positive control.

### Chromatin Immunoprecipitation (ChIP) and Quantitative PCR (qPCR)

ChIP experiments were conducted, as described previously, with minor modifications (Yamaguchi et al., 2014). Roots of five plants were pooling for one replication and three replication were used (*n* = (3). Each sample was immediately immersed in 1X phosphate buffered-saline (PBS). Roots were vacuum infiltrated with 1% formaldehyde in PBS for 15 min. Crosslinking reactions were quenched with 0.125 M glycine, and samples were washed with cold PBS. Samples were then ground in liquid nitrogen and solubilized in nuclei lysis buffer ([50 mM HEPES [(pH 7.5]), 150 mM NaCl, 1% Triton X-100, 0.1% sodium deoxycholate, 0.1% SDS, and protease inhibitor).]. Chromatin was sheared to 0.1–1 kb fragments using a sonicator (Covaris M220) at 10% amplitude and 4°C temperature. The sheared chromatin was harvested after centrifugation at 14,000 rpm for 10 min. The supernatant was pre-cleared with 40 μL Dynabeads Protein A/G (1001D, 1003D; Invitrogen) at 4°C for 2 h with rotation. The pre-cleared samples were incubated overnight with 2 μL anti-H3k27me3 or anti-H3k36ac antibody (07-449, 07-540; Milipore). The supernatant was mixed with 40 μL Dynabeads Protein A/G for 4 h with rotation. The beads were washed with nuclei lysis buffer, followed by low-salt buffer ([20 mM Tris-Cl [(pH 8.0]), 150 mM NaCl, 2 mM EDTA, 1% Triton X-100, and 0.1% SDS),], high-salt buffer ([20 mM Tris-Cl [(pH 8.0]), 500 mM NaCl, 2 mM EDTA, 1% Triton X-100, and 0.1% SDS),], LNDET buffer ([0.25 M LiCl, 1% Nonidet Non-idet P-40, 1% deoxycholic acid, 1 mM EDTA, and 10 mM Tris-Cl [(pH 8.0]))], and TE buffer (pH 8.0). Samples were eluted twice using 100 (μL elution buffer (0.1 M NaHCO3 and 1% SDS) for 30 min at 65°C. The eluates were incubated overnight with 12 (μL 5 M NaCl and 1 (μL protease K at 65°C for reverse crosslinking. Finally, the samples were purified using MinElute Reaction Cleanup Kit (28204; Qiagen) for ChIP-seq analysis. To determine gene expression, 0.3 μg purified DNA and 2 μg input DNA (control) were subjected to qPCR using sequence-specific primers (Supplementary Table S6).

### ChIP-Seq and Data Analysis

Genomic DNA libraries (insert size: 250–400 bp) were generated from the input and immunoprecipitated DNA and sequenced using Illumina HiSeq2500 to generate 100 bp paired-end reads. The sequenced reads were aligned to the *Arabidopsis* reference genome sequence (TAIR10) using Bowtie2 (v.2.2.2). The identification, estimation, and annotation of ChIP-seq peaks were carried out using the Homer (v.4.10) platform (Zhang et al., 2008).

### Total RNA Isolation and qPCR

The roots of five plants were pooled together for each replicate (*n* = 3–4). The harvested roots were immediately washed with water to remove soil and other debris, and frozen in liquid nitrogen. Total RNA extraction and qPCR were conducted, as described previously, with a minor modification (Tata et al., 2016). The primers used in qPCR analysis are listed in Supplementary Table S6.

### RNA-Seq and Data Analysis

Total RNA was isolated from *Arabidopsis* roots using the RNeasy Mini Kit (Qiagen), according to the manufacturer’s instructions. The quality and integrity of total RNA were confirmed by agarose gel electrophoresis and ethidium bromide staining, followed by a visual examination of RNA under ultraviolet light. RNA-seq library was prepared using the TruSeq RNA Sample Prep Kit v2 (Illumina, San Diego, CA, United States) and sequenced on the Illumina Hiseq2500 platform to generate 100 bp paired-end reads. Reads were quantified and mapped to the *Arabidopsis* reference genome (TAIR10) using the STAR software (Dobin et al., 2013). Differentially expressed genes (DEGs) were selected from the RNA-seq count data using the edgeR package (Robinson et al., 2010). Counts per million (CPM) mapped reads in each sample were used to estimate the expression level of each gene. Enrichment analysis was performed using DAVID, and data were visualized using ReviGO (*p* < 0.05).

### Small RNA-Seq and Data Analysis

Small RNA-seq libraries were constructed as described previously (Lu et al., 2007), with minor modifications. Total RNA was isolated using the mirVana miRNA Isolation Kit (Ambion). Small RNAs (20–30 nt) were separated on a 15% Novex TBE-Urea gel (Invitrogen) and purified. The purified small RNAs were ligated first with the 5′ RNA adapter and then with the 3′ RNA adapter provided in the TruSeq Small RNA Library Prep Kit (Illumina). At each step, the ligated product was subjected to polyacrylamide gel electrophoresis (PAGE) and gel purified. After first-strand synthesis and 11 cycles of PCR amplification, the product was separated by PAGE, gel purified, and submitted for sequencing on the Illumina NextSeq500 platform. Adapter sequences were removed from the raw sequences using Trimommatic (v.0.33), and then the cleaned sequences were mapped onto the *Arabidopsis* reference genome (TAIR10) using bowtie2 (v.2.2.2). The quantification and statistical analysis of mapped reads were carried out using HTSeq (Kim et al., 2013; Anders et al., 2015) and edgeR (Robinson et al., 2010) packages, respectively.

### Prediction of miRNA Targets and Generation of Heatmaps

Targets of the identified miRNAs were predicted based on the *Arabidopsis* reference genome (TAIR10 from the JGI genomic project, Phytozome 11) using target prediction software.^1^ Targets with < 3 expectation were selected according to the following parameters: (1) less than two mismatches in the seed region (2–13 nt) between miRNA and the target; (2) a penalty of 0.5 for G–U mismatches and of 1 for other mismatches; (3) a penalty of 1.5 for extra weight in the seed region; and (4) a penalty of 2 and 0.5 for opening gap and extending gap, respectively. Heatmaps for miRNAs and target genes were constructed using the R software (version 3.5.2).

### Lignin Quantification

Lignin was quantified as described previously (Schenk and Schikora, 2015), with a minor modification. Approximately 25–50 *Arabidopsis* roots (3–9 mg) per treatment were harvested and freeze-dried for 1 day. One milliliter of 80% methanol was added to the samples, followed by incubation at 25°C for 1 h. Next, the samples were centrifuged at 1,300 × *g* for 10 min, and supernatants were discarded. The pellets were washed with 1 mL 80% methanol, followed by SDW and acetone. To conduct alkaline hydrolysis, the pellets were incubated in 1 mL 1 M NaOH at 80°C for 1 h and subsequently overnight at room temperature. On the next day, 100 (μL of 86% phosphoric acid and 500 L of ethyl acetate were added to the samples and incubated on a rotary shaker at 25°C for 30 min. The samples were centrifuged at 1,300 × *g* for 5 min, and supernatants were discarded. To extract lignin, 500 μL of 80% methanol was added to the pellets, and the samples were centrifuged at 1,300 × *g* for 10 min. The pellets were first washed with 1 mL 80% methanol, followed by SDW and acetone, and then dried in a SpeedVac for 10 min. Next, 1.5 mL 2 M HCl and 0.3 mL thioglycolic acid were added to the dried pellets, and the samples were incubated on a shaker at 95°C for 4 h. Samples were cooled briefly on ice and then centrifuged at 13,000 × *g* for 5 min. The pellets were washed with SDW twice and centrifuged at 13,000 × *g* for 10 min. The residues were incubated with 1 mL 0.5 M NaOH overnight on a shaker. On the next day, samples were centrifuged at 13,000 × *g* for 5 min, and supernatants were collected. Then, 0.5 mL NaOH was added to the pellets, and samples were centrifuged at 13,000 × *g* for 5 min. Supernatants were combined and acidified with 300 μL 32% HCl. Samples were incubated on a shaker at 4°C for 4 h and then centrifuged at 13,000 × *g* for 5 min. The lignin pellet was dissolved in 100–200 μL 0.5 M NaOH. A standard curve (alkali, 2-hydroxy-proyl ether, Aldrich 370967) was generated for sample quantification, and lignin content was determined by measuring the absorbance at 340 nm and expressed as μg alkali lignin mg^–1^ dry root.

### Statistical Analysis

To estimate the statistical significance of differences in gene expression data obtained by RNA-seq or small RNA-seq, DEGs were identified based on count data using an EdgeR package that uses a negative binomial model. The gene count dispersion was estimated using the adjusted profile likelihood method of Cox and Reid. After model fitting and dispersion estimation, DEGs were selected using a generalized linear model (GLM) likelihood ratio test that specifies probability distributions according to the mean-variance relationship. The GLM likelihood ratio test is based on the principle of fitting negative binomial GLMs with Cox-Reid dispersion estimates. Expression level differences in genes were considered statistically significant if the *P-value* was < 0.05 and the fold difference in expression between two sample groups was ≥ 1.5. To detect differentially bound peaks between two sample groups in ChIP-seq data, we used the Homer software platform, which adopts a cumulative Poisson distribution. In this test, differences in ChIP-seq peaks were considered statistically significant if the Poisson enrichment *P-value* over the background tag count was 0.0001 and fold enrichment over the background tag count was 4. To identify significant gene sets associated with biological processes, functional enrichment analysis was conducted using the DAVID software, in which the significance of over-represented gene sets was estimated by Fisher’s Exact test (*P* < 0.05). The experimental data sets were subjected to the analysis of variance (ANOVA) using the JMP software (version 5.0; SAS Institute, Inc., Cary, NC, United States). Significant effects of treatments were determined based on the magnitude of the *F*-value (*P* = 0.05). When a significant F-test was obtained, separation of means was accomplished by Fisher’s protected least significant difference (LSD) test (*P* = 0.05).

## Results

### SV Treatment Triggers Induced Resistance Against *R. solanacearum* in Arabidopsis

**FIGURE 2:**
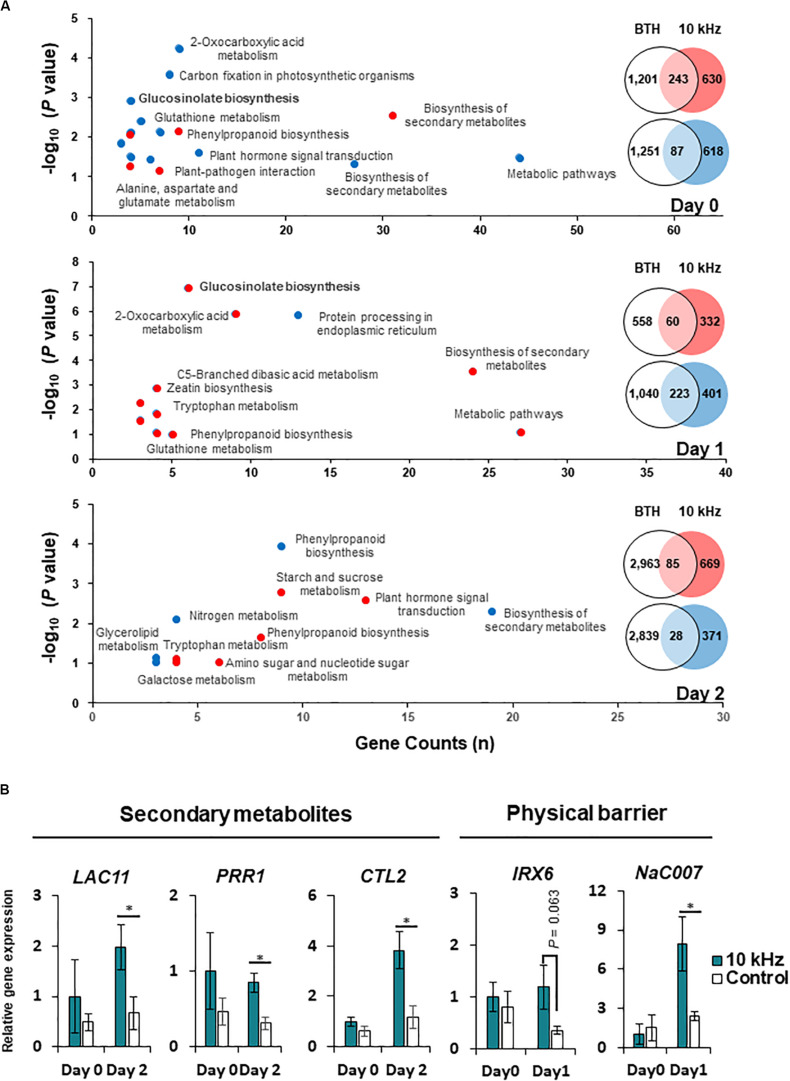
Modification of gene expression in SV (10 kHz)-treated *Arabidopsis* plants compared with BTH-treated plants. **(A)** Venn diagram and major functions of differentially expressed genes (DEGs) in 10 kHz-treated plants compared with BTH-treated plants at 0, 1, and 2 days post-inoculation (dpi) with *R. solanacearum*. Up-regulated genes are indicated in red, and down-regulated genes are indicated in blue. **(B)** Quantitative PCR (qPCR)-based validation of RNA-seq data. The expression of genes related to biosynthesis of secondary metabolites and physical barrier were examined by qPCR. (*n* = 3–4; *p < 0.05).

Aboveground plant parts of *Arabidopsis* seedlings were treated with six different SVs (0.2, 1, 5, 10, 15, and 20 kHz) each at 90 dB. Among these SV treatments, the 10 kHz treatment triggered the greatest resistance against *R. solanacearum* in roots, similar to that triggered by the chemical trigger benzothiadiazole (BTH; positive control) (Figures 1A,B and Supplementary Figure S1). We performed RNA-sequencing (RNA-seq) of 10 kHz and BTH treated *Arabidopsis* roots to investigate 10 kHz SV-specific transcriptional changes. The results of Kyoto Encyclopedia of Genes and Genomes (KEGG) pathway enrichment analysis of DEGs (10 kHz vs. BTH treatments) conferred plant immunity group including “plant-hormone signal transduction,” “plant-pathogen interaction,” and “phenylpropanoid biosynthesis” (Figure 2A and Supplementary Tables S1, S2). In comparison with DEGs between 10 kHz SV and BTH treatment, 10 kHz SV specific DEGs were detected (Figure 2A and Supplementary Table S1). The results of Kyoto Encyclopedia of Genes and Genomes (KEGG) pathway enrichment analysis revealed that 10 kHz SV potentiated plant immunity (defense priming) in *Arabidopsis* after inoculation with *R. solanacearum* (Figure 2A and Supplementary Table S1).

Next, we investigated how 10 kHz SV triggers induced resistance in plants. To determine the effect of 10 kHz SV on plants, we selected DEGs showing 1.5-fold change in expression (*p* (0.05) between 10 kHz-treated plants and control plants (Supplementary Figures S2A–C and Supplementary Table S3). Arabidopsis plants were exposed to 10 kHZ for 10 days and then inoculated with *R. solanacearum*. DEGs identified at 0, 1, and 2 days post-inoculation (dpi) were involved in “response to stimulus,” “phenylpropanoid catabolism,” and “cell wall organization or biogenesis,” respectively (Supplementary Figures S2D–F). These RNA-seq data were verified by qPCR (Figure 2B). The expression of phenylpropanoid biosynthetic genes, *LAC11* (AT5G03260), *PRR1* (AT1G32100), and *CTL2* (AT3G16920), increased in 10 kHz treated plants at 2 dpi compared with the control. Additionally, the qPCR results showed that the expression of secondary cell wall biosynthesis genes, *IRX6* (AT5G15630) and *NAC007* (AT1G12260), in SV-treated plants increased at 1 dpi, corroborating the RNA-seq data. These results indicate that activation of external reaction (0 dpi) by SV might lead to defense activation-like secondary metabolites (1 dpi) and physical barrier (2 dpi).

### SV-Triggered Induced Resistance Mediates H3K27me3 Modification

**FIGURE 3:**
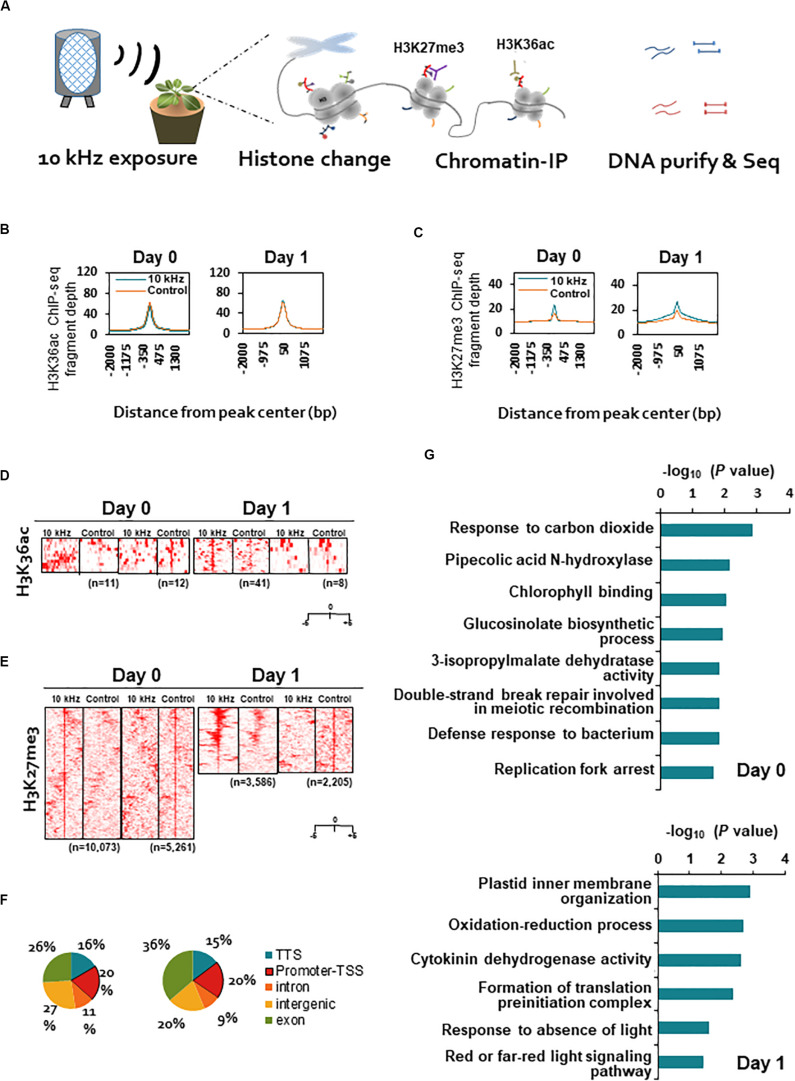
Genome-wide profiling of H3K36ac and H3K27me3 modifications in SV-treated plants during *R. solanacearum* infection. **(A)** Schematic representation of the experimental design used for chromatin immunoprecipitation (ChIP)-seq analyses of H3K36ac and H3K27me3 modifications in 10 kHz-treated and control plants at 0 and 1 dpi. **(B,C)** Fragment depth from peak center of H3K36ac **(B)** or H3K27me3 **(C)**. **(D,E)** Heatmaps of ChIP-seq signals at ± 5 kb around the peak midpoints of H3K36ac **(D)** or H3K27me3 **(E)**. **(F)** Distribution of H3K27me3 sites within genic regions. A 1 kb region upstream of the transcription start site (TSS) was defined as the promoter. **(G)** Gene ontology (GO) analysis of genes carrying the H3K27me3 mark in promoter regions.

In many case studies, induced resistance is coupled with defense priming involving epigenetic gene regulation (Park, 2009; Espinas et al., 2016). Therefore, we investigated two histone modifications as potential SV effectors, H3K36ac (transcription activation mark) and H3K27me3 (transcription repression mark), using ChIP-seq (Figure 3A). Significant differences were detected in the abundance of H3K27me3 modification, but not in that of H3K36ac modification, between 10 kHz-treated and control plants (Figures 3B–E). Next, to understand the functions of genes carrying the H3K27me3 mark in their promoter regions (total 174 genes), we performed gene ontology (GO) analysis (Figures 3F–G and Supplementary Table S4). The results revealed differential enrichment of histone modifications in the promoter regions of defense-related genes categorized as “glucosinolate biosynthetic process” and “cytokinin dehydrogenase activity” (Boivin et al., 2016; Liu et al., 2016; Figure 3G). Histone modification-mediated chromatin remodeling is one of the key epigenetic gene regulation strategies used to induce defense gene priming (Espinas et al., 2016). Therefore, H3K27me3 modification in the promoter regions of defense-related genes may lead to defense gene priming and adequate elicitation of induced resistance (an innate immune response).

### SV-Induced H3K27me3 Modification Affects GS Biosynthesis and Cytokinin (CK) Signaling Genes

To conduct an integrative comparison of ChIP-seq and RNA-seq data, we examined the expression of 174 H3K27me3-modified genes (Supplementary Tables S3, S4). A total of 39 genes showed H3K27me3-mediated transcriptional changes, of which 12 genes (30.7%) encoded membrane-localized proteins (Table 1). Given that external stimuli are recognized by sensors in the cell membrane (Kumar, 2018), it is possible that these membrane-localized proteins are affected by SV.

**TABLE 1:** Correlation between H3K27me3 modification and RNA-seq data in *Arabidopsis* plants treated with sound vibration (SV) of 10 kHz.

Gene^§^	H3K27me3^‡^	Log_2_FC^†^	*P-value	Description	Localization
*CA1*	+	–5.15	1.89E-06	Beta carbonic anhydrase 1	Plasma membrane, Chloroplast stroma
*AT2G36220*	+	–1.43	1.42E-08	Anaerobic respiration	–
***GSTF11***	+	–1.37	8.66E-05	Glutathione S-transferase F11, detoxification against herbicides	Cytosol
***IPMI2***	+	–1.35	2.44E-08	3-isopropylmalate dehydratase small subunit 1, glucosinolate biosynthetic process	Chloroplast stroma
*AT3G03280*	+	–1.22	1.39E-05	Uncharacterized	Transmembrane
***AT2G26695***	+	–0.98	2.19E-03	Metal ion binding, cytokinin responsive	–
*AT1G43910*	+	–0.92	2.99E-06	AAA-ATPase	Membrane
*ERF011*	+	–0.91	6.31E-08	Ethylene-responsive transcription factor ERF011	Nucleus
*AT1G20990*	+	–0.84	1.07E-02	Tubulin beta chain	Cytoskeleton
*AT5G11620*	+	–0.77	2.31E-03	Translation machinery-associated protein 22	Cytoplasm
*AT4G30450*	+	–0.72	7.40E-05	Glycine-rich protein	–
*AT2G21185*	+	–0.72	1.68E-04	Uncharacterized	Transmembrane
*MYB32*	+	–0.71	2.16E-04	Transcription factor MYB32	Nucleus
***MAM1***	+	–0.70	2.59E-03	Methylthioalkylmalate synthase 1, chloroplastic	Chloroplast
*AT3G11591*	+	–0.70	2.69E-02	Bric-a-brac protein	–
*SYP125*	+	–0.70	1.16E-02	SNAP receptor activity	Transmembrane
*FAD4L2*	+	–0.63	1.82E-03	Fatty acid desaturase 4-like 2, chloroplastic	Transmembrane (chloroplast)
*ALMT10*	+	–0.62	6.50E-04	Aluminum-activated malate transporter 10	Transmembrane
*AT2G32190*	+	–0.60	1.50E-02	Cysteine-rich/transmembrane domain A-like protein	Plasma membrane
*AT5G57100*	+	–0.59	2.02E-03	Nucleotide-sugar uncharacterized transporter 1	Transmembrane
*AT1G44414*	+	–1.45	4.07E-03	Uncharacterized	–
*GRXS10*	+	–1.01	2.06E-02	Monothiol glutaredoxin-S10	Nucleus
*AT4G31470*	+	–0.73	3.04E-02	CAP (Cysteine-rich secretory proteins, Antigen 5, and Pathogenesis-related 1 protein) superfamily protein	Extracellular space
*SCY1*	+	–0.59	6.60E-03	Preprotein translocase subunit SCY1, chloroplastic	Transmembrane (chloroplast)
*AT2G27660*	-	1.13	4.32E-04	Pectinesterase inhibitor activity	–
*ARFD1A*	-	1.12	5.39E-03	ADP-ribosylation factor D1A, transporter	Cytoplasm
*AT4G16960*	-	1.03	2.07E-03	Mitochondrial pyruvate carrier, Disease resistance protein (TIR-NBS-LRR class)	Transmembrane (Mitochondrion)
*AT5G42010*	-	0.98	8.64E-03	Transduction/WD40 repeat-like superfamily protein, uncharacterized	–
*AT1G13480*	-	0.95	2.16E-02	Proteasome subunit beta	Nucleus, cytoplasm
*PLP3*	-	0.89	6.25E-03	Patatin-like protein 3	Cytoplasm
*AT3G50350*	-	0.8	1.73E-03	Membrane insertase, putative (DUF1685)	–
*WRKY43*	-	0.80	1.91E-02	WRKY transcription factor 43, DNA binding	Nucleus
*AT4G15810*	-	0.76	2.31E-02	P-loop containing nucleoside triphosphate hydrolases superfamily protein	–
*WRKY54*	-	0.75	2.62E-03	Probable WRKY transcription factor 54	Nucleus
*AT4G23540*	-	0.73	2.23E-03	snRNA processing	Transmembrane, Nucleus
*SNL6*	-	0.64	2.63E-03	Paired amphipathic helix protein Sin3-like 6	Nucleus
*PRP39-2*	-	0.59	1.29E-02	Tetratricopeptide repeat (TPR)-like superfamily protein	Nucleus
***CKX1***	-	0.90	9.86E-03	Cytokinin dehydrogenase 1	Vacuole
*HTR2*	-	0.82	9.17E-03	Histone H3.2, nucleosomal DNA binding	Nucleus, Chromosome

^§^Bold font indicates glucosinolate (GS) biosynthesis or cytokinin (CK) signaling genes. ^‡^The + and - symbols indicate increased or decreased H3K27me3 modification determined from chromatin immunoprecipitation (ChIP) signals (2-fold difference; Poisson P < 0.01), respectively, in 10 kHz-treated plants compared with control plants. Genes with ± H3K27me3 modifications in promoter regions were identified from differentially expressed genes (DEGs) in RNA-seq data (1.5-fold change; p < 0.05). ^†^Relative gene expression in 10 kHz-treated plants compared with control is presented as Log2 fold change (Log2FC). *P-value was obtained from a generalized linear model (GLM) likelihood ratio test.

**FIGURE 4:**
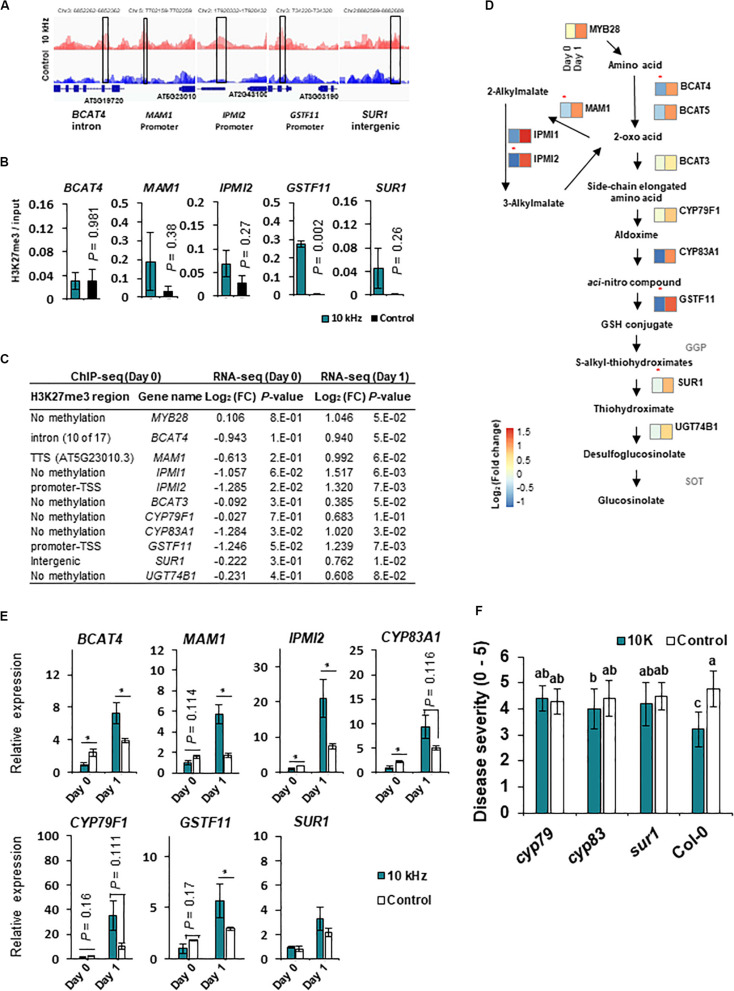
SV (10 kHz) modulates the expression of glucosinolate (GS) biosynthesis genes through chromatin remodeling**. (A)** Integrated Genome Browser (IGB) view of H3K27me3 modifications, as determined by ChIP-seq. Data represent the average of three biological replicates. Y-axis scale is identical for the different tracks. Blue diagrams indicate gene structure; boxes represent the regions tested in ChIP-qPCR experiments. **(B)** ChIP-qPCR validation (*n* = 3). **(C,D)** Expression of aliphatic GS biosynthesis genes. Heatmaps show expression levels of genes differentially expressed between 10 kHz-treated and control plants (*n* = 3–4). Asterisk indicates the enrichment of H3K27me3 modification in 10 kHz-treated plants vs. control plants. **(E)** Validation of GS biosynthesis gene expression by qPCR (*n* = 3–4; *p < 0.05). **(F)** Disease severity in 10 kHz-treated and control GS biosynthesis mutant plants at 14 dpi with *R. solanacearum*. Data represent mean ± SE. Different letters indicate significant differences [*n* = 10; *p* < 0.05; Fisher’s least significant difference (LSD) test].

Among the H3K27me3-modified genes, aliphatic GS biosynthesis-related genes, *MAM1*, *IPMI2*, and *GSTF11*, showed corresponding transcriptional changes (Figures 4A–D and Table 1). GSs are secondary metabolites in the Brassicaceae family with antimicrobial and antiherbivore properties (Appel and Cocroft, 2014; Sotelo et al., 2015; Liu et al., 2016). Therefore, we closely examined the expression of all GS biosynthesis genes (Figures 4C,D). Among the 14 GS biosynthesis genes, 12 were down-regulated at 0 dpi but up-regulated at 1 dpi (Figures 4C,E). Redundant H3K27me3 modification was detected in *GSTF11*, *IPMI2*, and *MAM1* promoters, *BACT4* intron, and *SUR1* intergenic region in SV-treated plants (Figures 4A–C). Thus, the expression of GS biosynthesis genes in SV-treated plants was tightly suppressed by the H3K27me3 modification at 0 dpi and subsequently released, suggesting SV-mediated epigenetic regulation of GS biosynthesis, thus triggering induced resistance. Similar to our results, Arabidopsis leaves pre-treated with caterpillar feeding-derived SV showed higher levels of aliphatic GS-mediated defenses than untreated plants when subsequently fed upon by *Pieris rapae* caterpillars (Appel and Cocroft, 2014). To investigate the effects of SV on the GS biosynthesis pathway, *cyp79*, *cyp83* and *sur1* mutants were treated with 10 kHz (Figure 4F and Supplementary Figure S4A). The disruption of upstream genes in the GS biosynthesis pathway mitigated the level of induced resistance. Thus, our results suggest that SV triggers the priming of GS-related genes in *Arabidopsis* for activating resistance against *R. solanacearum*.

**FIGURE 5:**
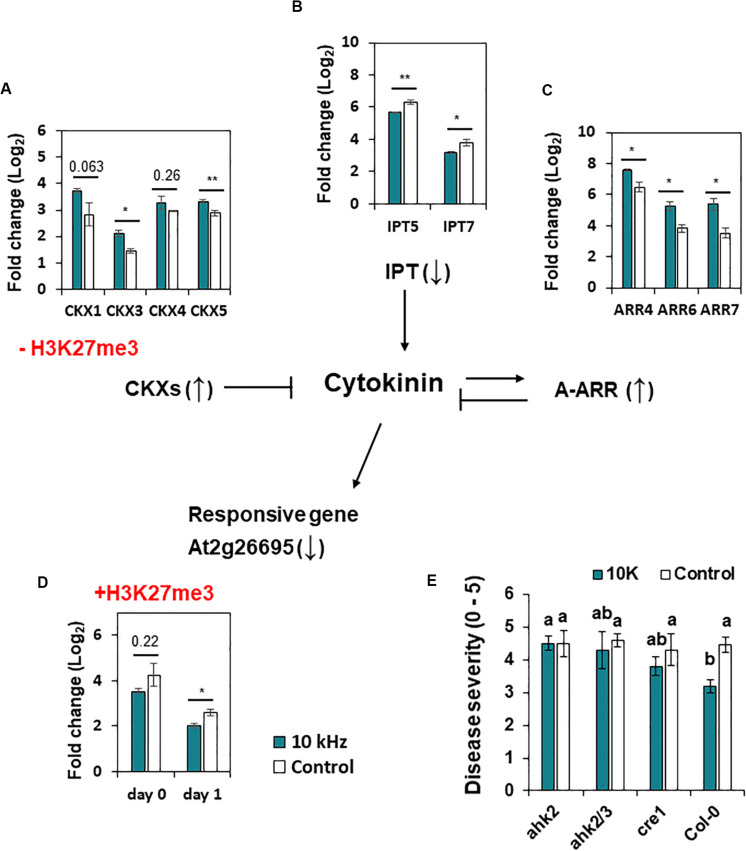
Expression of cytokinin (CK) signaling genes and H3K27me3 modification in *A. thaliana*. **(A–D)** Expression of *CK dehydrogenase/oxidase* (*CKX*) genes at day 1 **(A)**, *adenosine phosphate isopentenyl-transferase* (*IPT*) genes at day 0 **(B)**, *type-A Arabidopsis thaliana response regulator* (*ARR*) genes at day 0 **(C)**, and CK-responsive genes (*At2g26695*) **(D)** in 10 kHz-treated and control plants. *CXK1* and *At2g26695* showed reduced and enhanced H3K27me3 modification in 10 kHz-treated and control plants, respectively. Data represent mean ± SE (*n* = 3–4). Asterisks indicate significant differences (*p < 0.05; **p < 0.01; Student’s *t*-test). **(E)** Disease severity in 10 kHz-treated and control CK receptor mutant plants at 14 dpi with *R. solanacearum*. Data represent mean ± SE. Different letters indicate significant differences (*n* = 10; *p* < 0.05; Fisher’s LSD test).

We also identified H3K27me3 modification in CK signaling genes (Table 1). CK is a phytohormone that negatively regulates defense against root pathogens (Boivin et al., 2016). In our study, *CK oxidase/dehydrogenase* (*CKX*) genes (*CKX1*, *CKX3*, *CKX4*, and *CKX5*) and *type-A Arabidopsis response regulator* (*ARR*) genes (*ARR4*, *ARR6*, and *ARR7*; negative regulators of CK signaling) were up-regulated in 10 kHz-treated plants (Figures 5A,C), whereas *adenosine phosphate isopentenyltransferase* genes (*IPT5* and *IPT7*; CK biosynthesis genes) and a CK-responsive gene (*AT2G26695*) were down-regulated, thus inactivating CK signaling (Figures 5B,D; Bartrina et al., 2011). Among these genes, *CKX1* and *AT2G26695* were modulated by H3K27me3 modification (Table 1 and Figure 5). To confirm the relevance between CK signaling and SV-induced resistance, three CK receptor mutants, *ahk2*, *ahk2/3*, and *cre1*, were exposed to 10 kHz SV (Figure 5E and Supplementary Figure S4B). The *ahk2*, *ahk2/3*, and *cre1* mutants showed attenuated disease resistance. Given the overall tendency of the decline in CK signaling in 10 kHz-treated plants, our results imply that the inactivation of CK signaling by 10 kHz SV increases plant immunity against *R. solanacearum*.

### Activities of Defense Response-Associated Genes

Based on the significant relationship between defense priming and SV, we further sought to identify an association between defense response-associated genes and SV. While exploring significantly enriched bio-functions associated with SV (Figure 2A), we observed that many genes with defense response-associated functions were significantly plentiful (Supplementary Figure S3A). Many genes were up- or down-regulated at 0 dpi, and the number of defense response-associated genes decreased after SV treatment (Supplementary Figure S3B). Among the 174 genes carrying the H3K27me3 mark in their promoter regions at 0 dpi (Supplementary Table S4), *CA1* and *CEJ1* (Supplementary Figure S3C), involved in defense against *R. solanacearum*, were significantly inhibited by SV, indicating that epigenetic activity of H3K27me3 regulates defense response at the early stage of SV treatment.

### SV-Triggered Induced Resistance Is Accompanied by Changes in miRNA Expression

**FIGURE 6:**
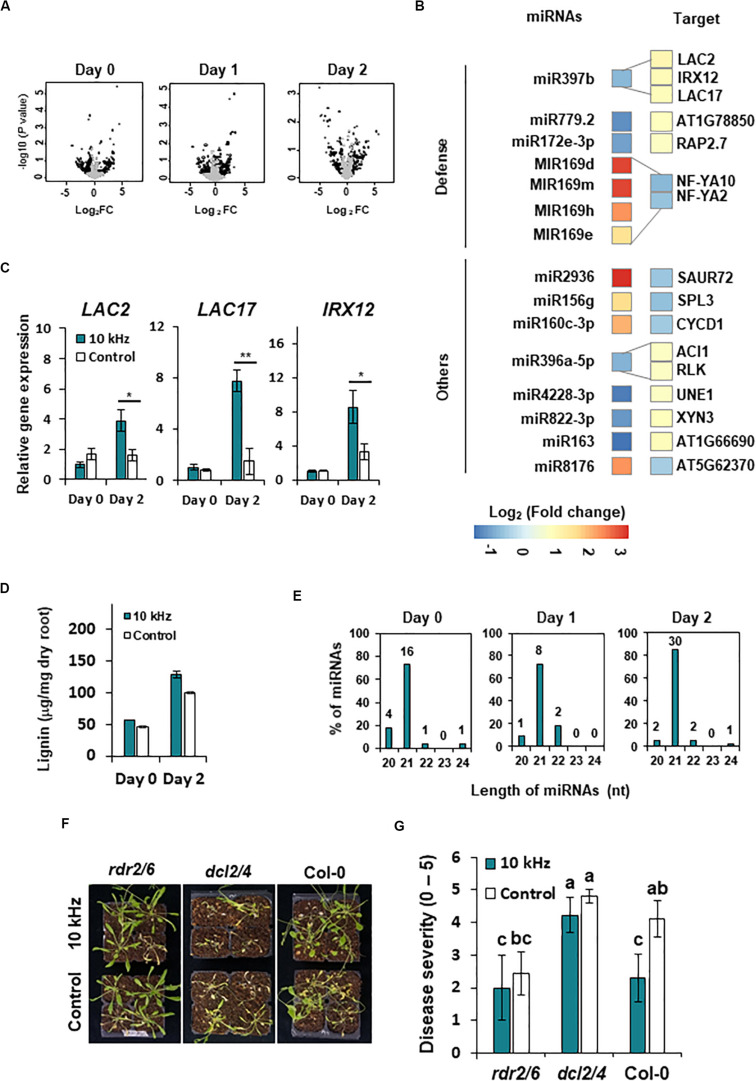
SV (10 kHz) elicits miRNA modulation in *Arabidopsis* plants inoculated with *R. solanacearum*. **(A)** Volcano plots of miRNAs differentially expressed (DE) between 10 kHz-treated and control plants. Black dots indicate significantly DE miRNAs (1.5-fold change; *p* < 0.05). **(B)** Heatmaps showing relative gene expression levels in 10 kHz-treated vs. control plants (1.5-fold change; *p* < 0.05). Relative expression levels (log_2_-transformed values) of miRNAs (left) and corresponding mRNAs (right) are indicated on a color scale. **(C)** Validation of *LAC2*, *LAC17*, and *IRX12* expression by qPCR. Data represent mean ± SE (*p < 0.05; **p < 0.01). **(D)** Quantification of lignin content. Data represent mean ± SE (**p < 0.01). **(E)** Length distribution of DE miRNAs. **(F,G)** Representative disease symptoms and disease severity in 10 kHz-treated and control plants at 14 dpi with *R. solanacearum*. Data represent mean ± SE. Different letters indicate significant differences (*n* = 10; *p* < 0.05; Fisher’s LSD test).

Similar to other epigenetic effectors, miRNAs modulate gene expression by post-transcriptional and transcriptional gene silencing (Vaucheret, 2006; Kaikkonen et al., 2011; Catalanotto et al., 2016). We performed miRNA-seq to identify miRNAs that regulate gene expression in 10 kHz-treated plants post-*R. solanacearum* inoculation. Differentially expressed (DE) miRNAs were compared with RNA-seq data to determine whether these miRNAs regulate gene expression (1.5-fold change; *p* < 0.05) (Figure 6A and Supplementary Table S5). A total of 15 DE miRNAs showed a negative correlation with the target mRNAs (Figure 6B and Table 2). Among these target mRNAs, lignin biosynthesis genes, *LAC2*, *LAC17*, and *IRX12 (LAC4*), were up-regulated, while *miR397b* was down-regulated in 10 kHz-treated plants (Figures 6B,C and Table 2). Lignin concentration in the roots was increased in plants exposed to 10 kHz SV (Figure 6D). Since lignin is a component of plant secondary cell walls, biotrophic pathogen-induced lignification is a conserved pre-formed defense mechanism in *Arabidopsis* (Chezem et al., 2017). Because *LAC4* and *LAC17* are involved in lignin polymerization, disruption of these genes leads to narrower roots with lower lignification (Zhao et al., 2013). The *R. solanacearum*-resistant tomato cultivar LS-89 contains a higher number of redundant lignin biosynthesis genes than the susceptible cultivar Ponderosa (Ishihara et al., 2012). Additionally, using the MiEAA software,^2^ we further investigated the functional annotations of 150 DE miRNAs (Supplementary Table S5). Among these 150 DE miRNAs, only two miRNAs (*ath-miR163* and *ath-miR398b-3p*) were enriched under the GO term “defense response to bacterium” (GO0042742), which was non-significant (*P* > 0.05; Fisher’s Exact test) (data not shown). Therefore, the 10 kHz-induced *LAC2*, *LAC17*, and *LAC4* activation observed in our study may have contributed to plant cell wall reinforcement, thus strengthening the barrier against *R. solanacearum* in *Arabidopsis* roots.

**TABLE 2:** Correlation between miRNA expression and RNA-seq data in 10 kHz-treated *Arabidopsis* plants.

miRNA^§^	Target genes	Log_2_FC^†^	*P*-value	Description
**Day 1**				
miR156g	AT2G33810 (SPL3)	–0.86	3.55E-04	Metal binding
miR4228-3p	AT1G29300 (UNE1)	0.64	1.71E-01	Intracellular protein transporter, putative (DUF641)
**Day 2**				
miR2936	AT3G12830 (SAUR72)	–0.74	3.79E-02	Auxin–responsive protein
MIR169d				
MIR169h	AT5G06510 (NF-YA10)	–0.95	3.80E-03	Nuclear transcription factor Y subunit A-10
MIR169m	AT3G05690 (NF-YA2)	–0.81	6.25E-03	Nuclear transcription factor Y subunit A-2, DNA binding
MIR169e				
miR8176	AT5G62370	–0.64	3.62E-02	Pentatricopeptide repeat-containing protein
miR160c-3p	AT1G70210 (CYCD1)	–0.69	2.79E-03	Cell cycle activation, embryonic root protrusion
miR396a-5p	AT5G01370 (ACI1),	0.64	1.37E-02	ACL-interacting protein fruit dehiscence
	AT2G34530 (RLK)	0.63	8.20E-03	Protein kinase
MIR397b	AT2G29130 (LAC2)	0.93	1.09E-02	Cell wall lignification
	AT2G38080 (IRX12)	0.82	7.06E-03	
	AT5G60020 (LAC17)	0.75	5.14E-02	
MIR163	AT1G66690,	0.76	9.56E-02	Paraxanthine methyltransferase 2/
	AT2G12480 (SCPL43)	0.76	9.56E-02	Serine carboxypeptidase-like 43
miR822-3p	AT4G08160 (XYN3)	0.74	2.81E-02	Endo-1,4-beta-xylanase 3
miR172e-3p	AT2G28550 (RAP2.7)	0.64	6.16E-02	Ethylene-responsive transcription factor RAP2-7
miR779.2	AT1G78850	0.69	2.82E-02	EP1-like glycoprotein 3

*^§^Differentially expressed (DE) miRNAs showing at least 1.5-fold change (p < 0.05) in expression were selected. Among the DE miRNA target genes, those that met the selection criteria (1.5-fold change; p < 0.05) are presented. ^†^Relative gene expression of 10 kHz-treated plants compared with control plants is presented as Log 2 fold change (Log2FC).*

Although 15 out of 120 DE miRNAs targeted host mRNAs, the functions of the remaining DE miRNAs were unknown. The majority of DE miRNAs identified at 0–2 dpi were 21 nt in length (Figure 6E and Supplementary Table S5). To obtain genetic evidence on the involvement of miRNAs in 10 kHz SV-mediated plant immunity, 21–22 nt miRNA processing mutants (*dcl2/4*) and 24-nt miRNA processing mutants (*rdr2-1/6-15*) were exposed to 10 kHz SV (Brosnan et al., 2007; Polydore and Axtell, 2018). Among these miRNA biogenesis mutants, induced resistance was abolished only in the *dcl2/4* mutant (Figures 6F,G), suggesting that 21–22-nt miRNAs affect 10 kHz-elicited induced resistance in *Arabidopsis* in a DCL2/4-dependent manner.

## Discussion

Previous research in plant immunity focused on chemical or biological materials for inducing disease resistance. In this study, we newly suggest that 10 kHz SV can be used as a physical trigger to induce plant resistance against *R. solanacearum*. Mostly, induced resistance is coupled with defense priming (Park, 2009; Espinas et al., 2016). The defense priming phenomenon enables plants to respond quickly and/or more strongly to each subsequent pathogen infection (Espinas et al., 2016). Therefore, we addressed two questions regarding the SV-mediated immune enhancement in *Arabidopsis*: (1) are SV-mediated transcriptional changes during pathogen infections accompanied by histone modifications?; and (2) are these changes regulated by miRNAs?

By exploring the H3K27me3 modification, we found changes in GS and CK pathway genes. Previously, GSs extracted from *Brassica* species showed direct antimicrobial activity against *Xanthomonas campestris* pv. *campestris* and *Pseudomonas syringae* pv. *maculicola* (Sotelo et al., 2015). In addition, the *walls are thin 1* (*wat1*) *Arabidopsis* mutant, which expresses GS biosynthesis genes to low levels in roots, shows enhanced susceptibility to *R*. *solanacearum* (Denance et al., 2013). Therefore, GSs seem to play an important role in the defense against *R. solanacearum* in *Arabidopsis*. In our results, GS biosynthesis genes showed a rapid increase in expression upon pathogen inoculation accompanied by H3K27me3 modification (Liu et al., 2016) (Figure 4). This indicates that the expression of GS biosynthesis genes is regulated epigenetically in SV-treated plants.

The plant hormone CK affects various processes during plant growth and development including cell division, shoot initiation, leaf senescence, and biotic and abiotic stress response (Kieber and Schaller, 2014). Moreover, CK plays a positive role in plant–microbe interactions, specifically symbiotic rhizobium nodulation (Gamas et al., 2017). However, CK accumulation in plants causes susceptibility to root pathogens such as *Agrobacterium tumefaciens*, *Plasmodiophora brassicae*, and *R. solanacearum* (Siemens et al., 2006; Hwang et al., 2010; Boivin et al., 2016). Overexpression of *CKX* genes in *Arabidopsis* increases resistance against *P*. *brassicae*, indicating the importance of CK in the progression of clubroot disease (Siemens et al., 2006). Similarly, the *M. truncatula* CK receptor mutant, *MTcre1*, showed enhanced resistance to *R*. *solanacearum*, supporting the negative role of CK in plants against *R*. *solanacearum* (Moreau et al., 2014). Therefore, epigenetic down-regulation of CK biosynthesis genes in SV-treated plants represents a key strategy for counteracting *R*. *solanacearum*.

Interestingly, we found that 12 out of 39 H3K27me3 modified genes at 0 dpi are localized in transmembrane (Table 1). Since the plants are sessile organisms, they have to cope with and adapt to various environments. In many cases, the environmental stress is detected by sensors located in the cell membrane (Kumar, 2018). In metalliferous soils, genes encoding transmembrane proteins play an important role in the environmental adaptation of plants (Sailer et al., 2018). Therefore, it seems that the genes related to the cell membrane have changed a lot in response to SV, which is the external stimulus in this study. Some cell membrane-related genes play a role in disease resistance (Vincent et al., 2017). For example, *glutamate receptor* (*GLR*) genes, which were differently expressed between treatments in our RNA-seq analysis, are known to activate defense-related genes by regulating Ca^2+^ signaling (Vincent et al., 2017; Nguyen et al., 2018) (Supplementary Table S3). Our results also showed that *AT4G16960*, which is annotated as an *NLR* gene, is up-regulated by the reduction in H3K27me3 modification in SV-treated plants (Table 1). The NLR proteins are well-known plant membrane receptors that recognize pathogen-derived molecules and trigger immune responses (Qi and Innes, 2013). These results suggest the possibility that SV-mediated changes in transmembrane protein-encoding genes at 0 dpi lead to further intracellular responses against pathogen infection.

Plant cell wall is the first barrier against external stresses (Liu et al., 2018). Since lignin is a component of secondary cell wall in plants, increased accumulation of lignin strengthens the physical barrier, which minimizes the spread of pathogens, thus enhancing plant immunity (Liu et al., 2018). Studies show that the expression of lignin biosynthesis genes is up-regulated in plants infected with various pathogens such as necrotrophic bacteria, biotrophic bacteria, and fungi (Bonello et al., 2003; Ponce de Leon et al., 2007; Chezem et al., 2017). In potato (*Solanum tuberosum*) and tomato, *R*. *solanacearum*-resistant cultivars contain higher lignin content than susceptible cultivars (Ishihara et al., 2012; Ferreira et al., 2017). Therefore, SV-induced *LAC2, LAC17*, and *IRX12* activation in our study possibly contributed to plant cell wall reinforcement, thus strengthening the barrier against *R. solanacearum* in *Arabidopsis* root.

Among the DE miRNAs, the miR169 family members and their target genes, *NF-YA*, are known to regulate biotic and abiotic stress response and plant growth. The negative defense role of miR169 is reported in *Arabidopsis* and *Oryza sativa* against responsive *R. solanacearum and Magnaporthe oryzae* (Hanemian et al., 2016; Li et al., 2017). Abiotic stresses such as low phosphate, low nitrogen, and high sucrose induce *NF-YA2* and *NF-YA10* expression, indicating *NF-YA* is important to acclimate to abiotic stresses (Leyva-González et al., 2012). In addition, transgenic plants of miR169abc or miR169defg with increased *NF-YA* expression showed smaller rosettes and shorter primary roots (Sorin et al., 2014). Although our results showed increased accumulation of miR169 family members, and reduced *NF-YA2* and *NF-YA10* expression in SV-treated plants (Figure 6B), we speculate that the reduced *NF-YA2* and *NF-YA10* may be associated with adaptations, such as changing root architecture, for improving abiotic stress tolerance in an SV-dependent manner, rather than a response to pathogenesis.

In some plant species, miRNAs also regulate the mRNA of associated pathogens (Zhang et al., 2016; Liu et al., 2017; Wang et al., 2018). For instance, in cotton (*Gossypium arboretum*), miR398 and miR2950 directly target multiple open reading frames of the cotton leaf curl disease (CLCuD) virus, leading to translation inhibition and reduced disease severity (Akmal et al., 2017). Additionally, miR159 and miR166 target the mRNA of virulence genes encoding C-15 hydroxylase (HiC-15) and Ca^2+^-dependent cysteine protease (Clp-1), respectively, conferring resistance to *Verticillium dahliae* (Zhang et al., 2016). In the current study, we showed enhanced miR398b, miR398b-5p, and miR398b-3p expression in SV-treated plants on day 2 (Supplementary Table S5). However, further research is needed to investigate whether the SV-induced plant miRNAs target *R*. *solanacearum* genes.

Overall, we confirmed that 10 kHz SV acts as a physical trigger and elicits induced resistance against *R. solancearum* in *Arabidopsis* roots as well as transcriptional changes different from those caused by the chemical trigger BTH. Direct comparison of RNA-seq, ChIP-seq, and miRNA-seq data revealed the epigenetic response of GS biosynthesis, CK signaling, and lignin biosynthesis genes to 10 kHz SV. These data, therefore, support the role of a physical trigger in the epigenetic regulation of plant innate immunity. Taken together, our findings suggest that SV modulates the epigenetic regulation of genes involved in secondary metabolite biosynthesis, hormone signaling, and cell wall biosynthesis, thus enhancing induced resistance against *R. solanacearum*.

## Data Availability Statement

Sequencing data described in this manuscript have been deposited in the NCBI Gene Expression Omnibus (GEO) under the accession number GSE133325.

## Author Contributions

JJ designed the study, performed most of the experiments, interpreted the results, and wrote the manuscript. S-KK analyzed and interpreted the RNA-seq, miRNA-seq, and ChIP-seq data. S-HJ performed the mutant and qRT-PCR validation experiments. M-JJ designed the study. C-MR designed the study, interpreted the results, and wrote the manuscript. All authors contributed to the article and approved the submitted version.

## Conflict of Interest

The authors declare that the research was conducted in the absence of any commercial or financial relationships that could be construed as a potential conflict of interest.

## Footnotes

1 http://plantgrn.noble.org/psRNATarget

2 https://ccb-compute2.cs.uni-saarland.de/mieaa2/

## Supplementary Material

FIGURE S1 Optimization of SV-triggered induced resistance in *Arabidopsis thaliana* plants against *Ralstonia solanacearum*. **(A,B)** Phenotype and disease severity in SV- (0.2, 1, 5, 10, 15, or 20 kHz) or BTH-treated plants and control plants at day 14. Disease severity was scored as 0 (no symptoms), 1 (< 50% of rosette leaves wilted), 2 (< 100% of rosette leaves wilted), 3 (100% of rosette leaves wilted), 4 (100% of rosette leaves wilted and stems partially wilted), and 5 (complete plant collapse). The 10 kHz (dark green) SV- and BTH-treated plants showed statistically reduced disease severity compared with control plants (gray) (^∗∗^*p* < 0.01).

FIGURE S2 Transcriptomic profiling of 10 kHz-treated *A. thaliana* roots challenged with *R. solanacearum*. **(A–C)** Heatmaps of genes differentially expressed between 10 kHz-treated and control plants at 0 **(A)**, 1 **(B)**, and 2 **(C)** days post-inoculation (dpi; 1.5-fold change; *p* < 0.05). **(D–F)** Gene Ontology (GO) enrichment analyses of DEGs identified at 0 **(D)**, 1 **(E)**, and 2 **(F)** dpi.

FIGURE S3 Gene set enrichment analysis and expression pattern of genes involved in defense response elicited by SV. **(A)** Gene set enrichment analysis of genes involved in defense response significantly associated with SV. **(B)** Heatmaps displaying expression patterns of defense response genes whose expression levels were highly correlated with SV. **(C)** Comparison of *CA1* and *CEJ1* expression levels between control and 10 KHz SV treatments. CPM, count per million mapped reads.

FIGURE S4 Representative disease symptoms of 10 kHz-treated and control plants at 14 dpi with *R. solanacearum*. **(A,B)** Disease symptoms of 10 kHz-treated and control glucosinolate (GS) biosynthesis mutant plants **(A)** and cytokinin receptor mutant plants **(B)**.

TABLE S1 Differentially expressed genes (DEGs) identified between sound vibration (SV; 10 kHz)- or benzothiadiazole (BTH)-treated plants vs. control plants.

TABLE S2 Functional analysis of genes up- or down-regulated in 10 kHz-treated plants compared with control plants.

TABLE S3 Normalized gene expression levels in 10 kHz-treated and control plants determined by RNA-seq.

TABLE S4 Differential H3K27me3 enrichment in 10 kHz-treated vs. control plants.

TABLE S5 Differentially expressed (DE) miRNAs identified between 10 kHz-treated and control plants.

TABLE S6 List of primers used in this study.
