# Supplementary figures and images for: Sound Vibration-Triggered Epigenetic Modulation Induces Plant Root Immunity Against Ralstonia solanacearum
Source: Front Microbiol. 2020 Aug 21;11:1978. doi: 10.3389/fmicb.2020.01978 (PMC7472266; doi:10.3389/fmicb.2020.01978)

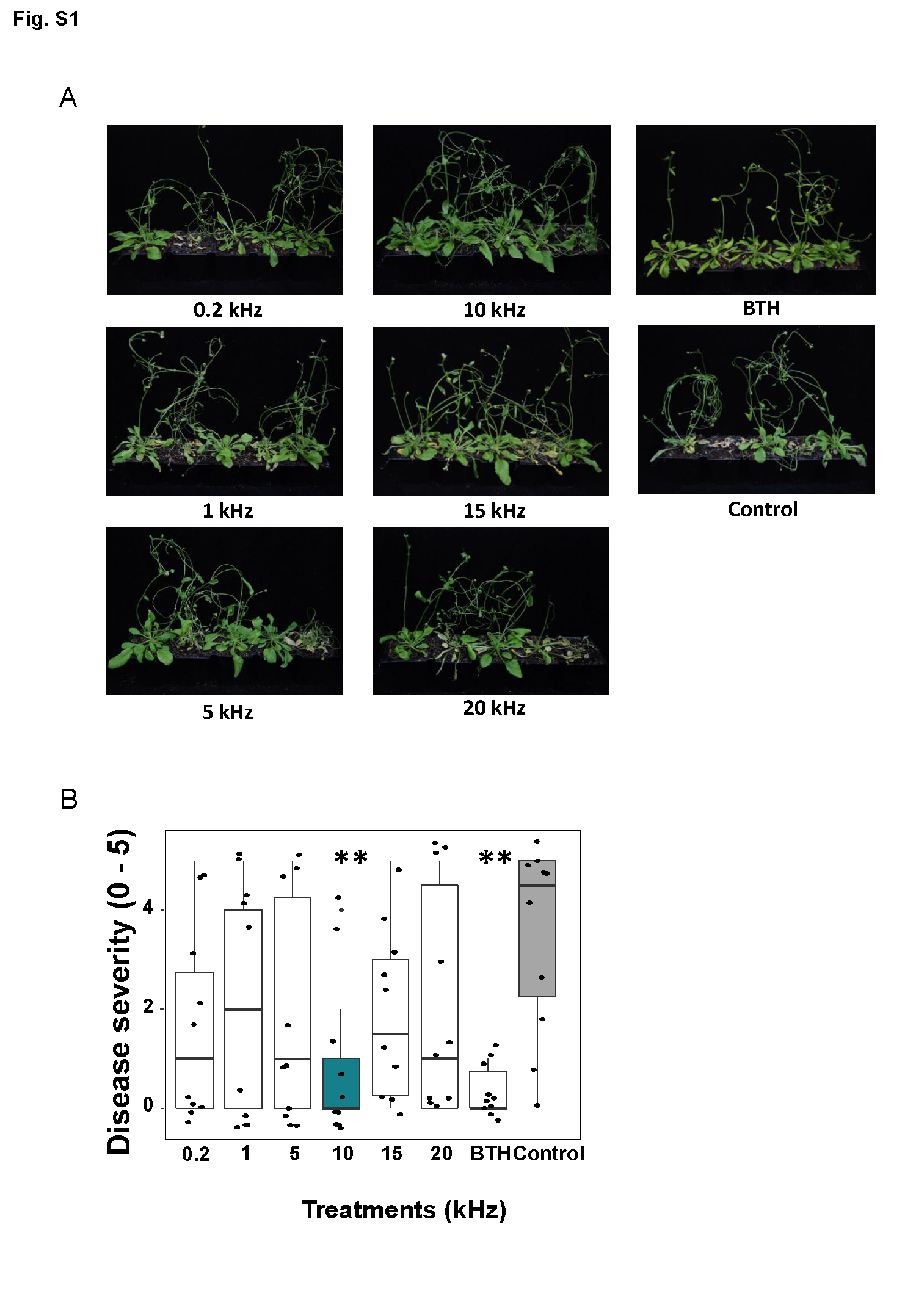

Supplement: FIGURE S1 — Optimization of SV-triggered induced resistance in Arabidopsis thaliana plants against Ralstonia solanacearum. (A,B) Phenotype and disease severity in SV- (0.2, 1, 5, 10, 15, or 20 kHz) or BTH-treated plants and control plants at day 14. Disease severity was scored as 0 (no symptoms), 1 (< 50% of rosette leaves wilted), 2 (< 100% of rosette leaves wilted), 3 (100% of rosette leaves wilted), 4 (100% of rosette leaves wilted and stems partially wilted), and 5 (complete plant collapse). The 10 kHz (dark green) SV- and BTH-treated plants showed statistically reduced disease severity compared with control plants (gray) (∗∗p < 0.01). [file Image_1.tiff]

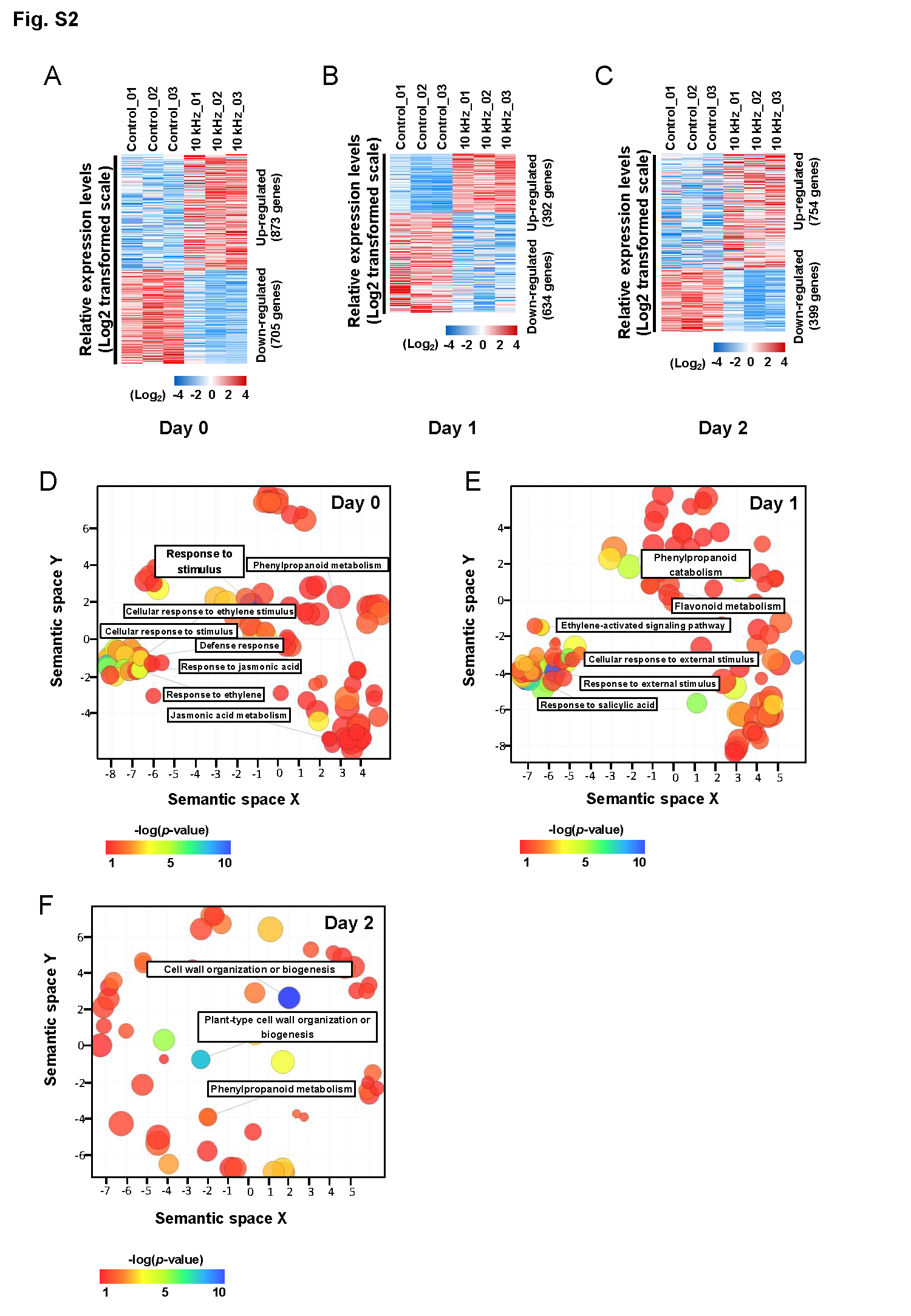

Supplement: FIGURE S2 — Transcriptomic profiling of 10 kHz-treated A. thaliana roots challenged with R. solanacearum. (A–C) Heatmaps of genes differentially expressed between 10 kHz-treated and control plants at 0 (A), 1 (B), and 2 (C) days post-inoculation (dpi; 1.5-fold change; p < 0.05). (D–F) Gene Ontology (GO) enrichment analyses of DEGs identified at 0 (D), 1 (E), and 2 (F) dpi. [file Image_2.tif]

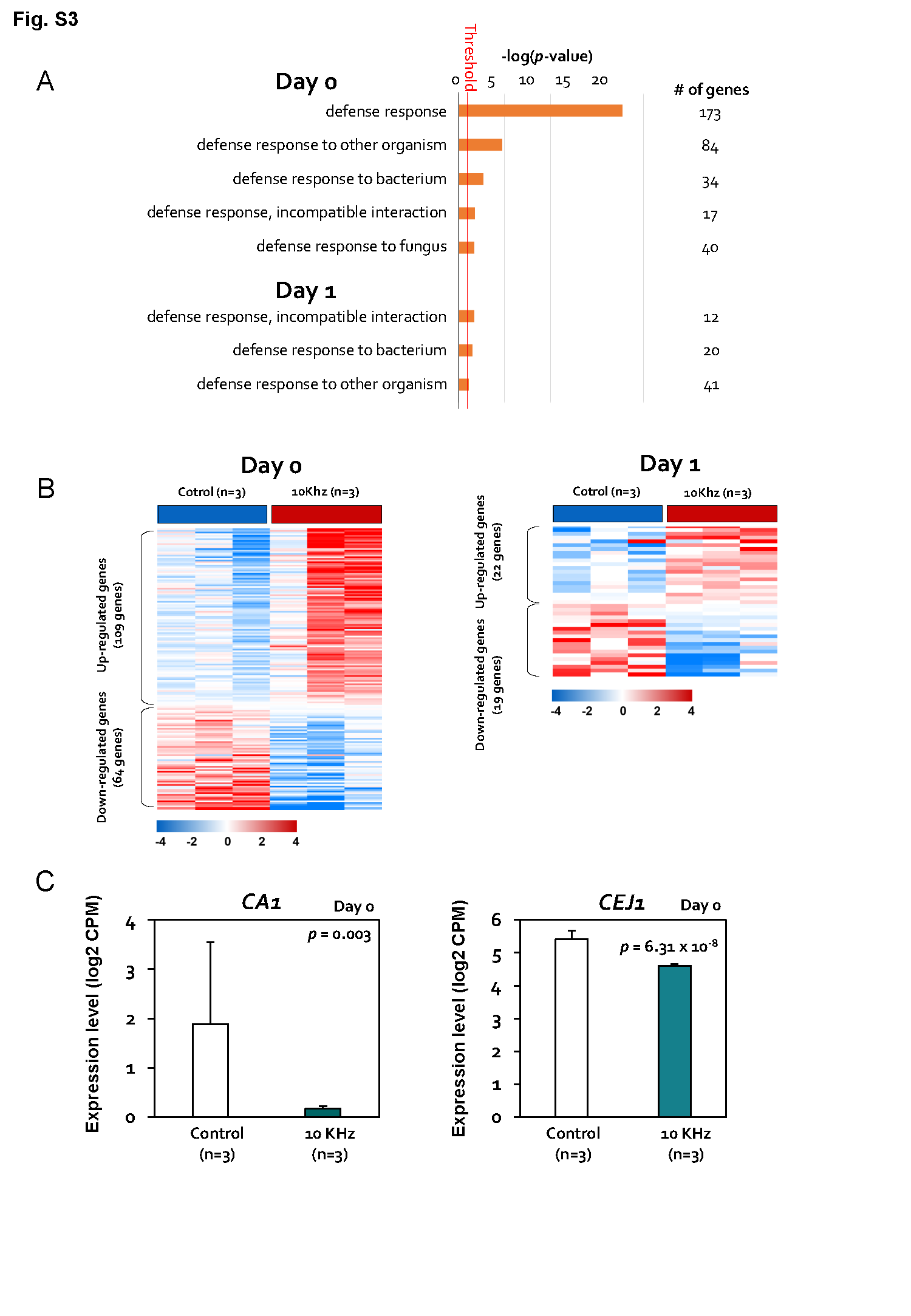

Supplement: FIGURE S3 — Gene set enrichment analysis and expression pattern of genes involved in defense response elicited by SV. (A) Gene set enrichment analysis of genes involved in defense response significantly associated with SV. (B) Heatmaps displaying expression patterns of defense response genes whose expression levels were highly correlated with SV. (C) Comparison of CA1 and CEJ1 expression levels between control and 10 KHz SV treatments. CPM, count per million mapped reads. [file Image_3.tif]

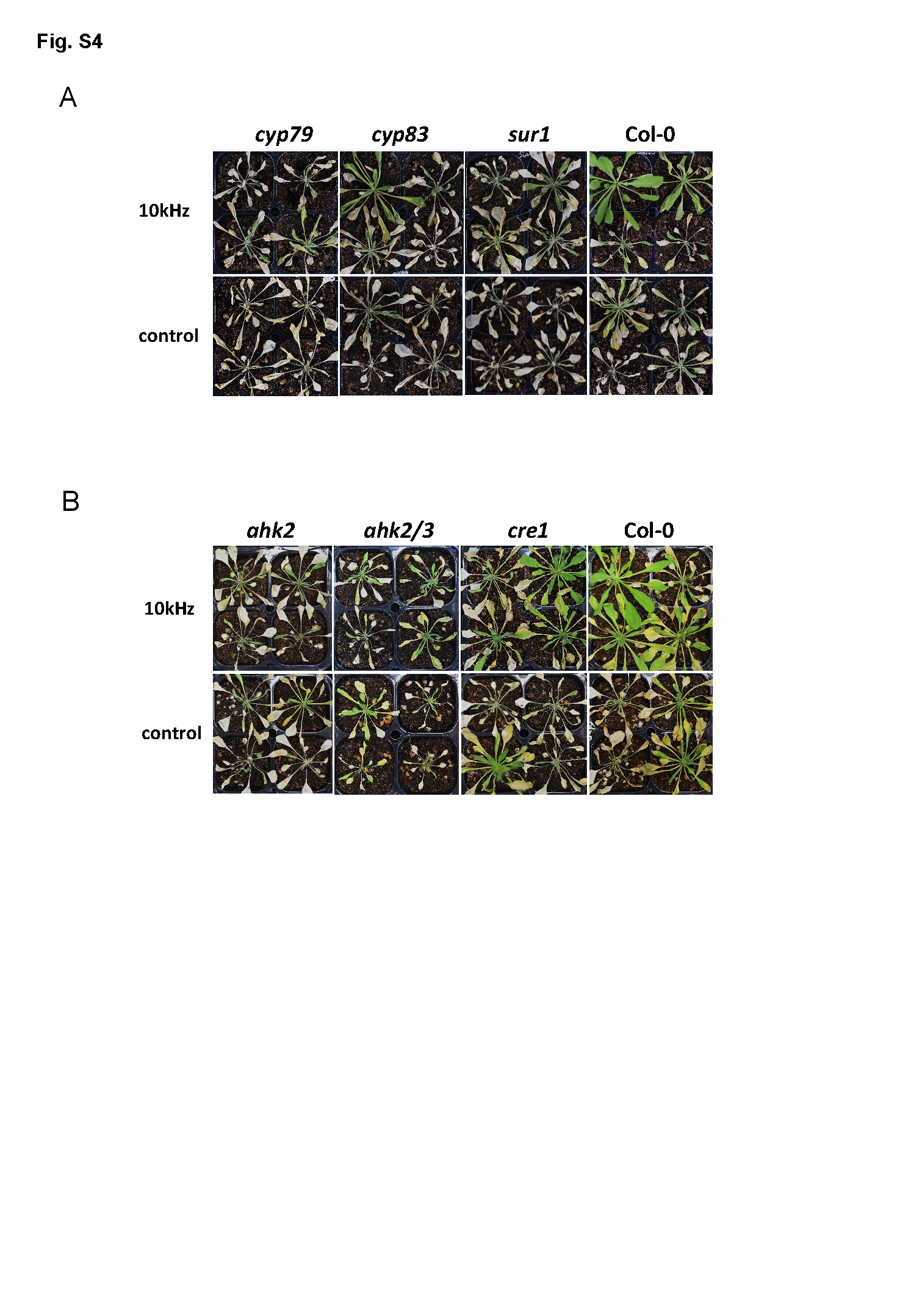

Supplement: FIGURE S4 — Representative disease symptoms of 10 kHz-treated and control plants at 14 dpi with R. solanacearum. (A,B) Disease symptoms of 10 kHz-treated and control glucosinolate (GS) biosynthesis mutant plants (A) and cytokinin receptor mutant plants (B). [file Image_4.tif]
